# Skilled birth attendant utilization trends, determinant and inequality gaps in Ethiopia

**DOI:** 10.1186/s12905-022-01995-5

**Published:** 2022-11-22

**Authors:** Workagegnhu Tarekegn, Sitota Tsegaye, Yemane Berhane

**Affiliations:** 1 Department of Nutrition and Behavioral Science, Addis Continental Institute of Public Health, Addis Ababa, Ethiopia; 2 Department of Epidemiology and Biostatistics, Addis Continental Institute of Public Health, Addis Ababa, Ethiopia

**Keywords:** Skilled birth attendant, Equality, Household wealth, Maternal education, Residency area, Ethiopia

## Abstract

**Background:**

Globally over half a million women die every year from potentially preventable and treatable pregnancy and childbirth complications; of which 99% occur in low-and middle-income countries (LMICs). The utilization of skilled birth attendants can timely identify treatable birth complications and save lives. However, utilization of services remained low in LMICs. This study aimed to examine the trends in the utilization of skilled birth attendants and the inequality gaps in Ethiopia using data from the Demographic and Health Surveys.

**Methods:**

We used data from five rounds of Ethiopian Demographic and Health Surveys conducted in the period 2000–2019. Respondents were women in the reproductive age group who had a live birth within five years preceding the surveys. We used the concentration curve and concentration index to identify the inequalities using the World Health Organization recommended Health Equity Analysis Toolkit software. We did a logistic regression analysis to examine factors associated with skilled birth attendant utilization using STATA version 14.0.

**Result:**

The skilled birth attendant coverage trend showed an increment from 5.7% in 2005 to 49.8% in 2019. The inequality gaps within the wealth, residence and education categories also showed a reduction over time. The odds of utilizing SBA were higher among those having primary, secondary, and above education status [AOR = 1.61 95%CI (1.33, 1.95)], being in the upper wealth quintile [AOR = 3.46 95%CI (1.8, 4.31)] and living in urban areas [AOR = 3.53 95%CI (1.88, 6.64)].

**Conclusion:**

The skilled birth attendant coverage trend showed a steady increase from 2005 to 2019 but if we continue with the current pace, it will be difficult to achieve the national target. The inequality gaps in household wealth status and residency area remain high. Efforts like strengthening the health system and engaging multisectoral agents need to be given priority to further reach the poorest and those living in rural areas to achieve national and international targets.

## Background

The presence of skilled attendants has a positive impact on the reduction in improving birth outcomes by facilitating early detection and management of complications during the birth process [1]. Lack of skilled birth attendance is considered one of the main challenges in improving pregnancy outcomes in low-income countries [2]. Which indicate the increase in the availability of skilled birth attendant during childbirth is a key intervention for preventing maternal and newborn deaths [3–5]. If a woman dies during childbirth, the survival of her infant and other children will be threatened. Infants without a mother are more likely to die within two years of his/her age, according to the World Health Organization (WHO), the International Confederation of Midwives (ICM), and the International Federation of Gynecology and Obstetrics (FIGO) skilled birth attendant is “an accredited health professional—such as a midwife, doctor or nurse—who has been trained to proficiency in the skills needed to manage uncomplicated pregnancies, childbirth and the immediate postnatal period, and in the identification, management and referral of complications in women and newborns [6, 7]”.

Every year over half a million women die because of pregnancy and childbirth complications globally [8, 9] and most maternal deaths are related to direct obstetric causes [9–12] from which up to 98 percent are preventable and treatable [8, 11, 13]. Most pregnancy complications occur in the process of birth and are often difficult to predict in advance, thus the presence of skilled attendants before, during, and immediately after childbirth is critically averting the unnecessary death of the mother and her newborn [3, 12].

The utilization of skilled birth attendants has been steadily increasing in low-income countries although service utilization is not universal across the social strata of any given society or country [1, 4]. Many studies showed that maternal educational status [6, 9, 12], age of the mother [14], previous pregnancy complication [9, 12, 15], accessibility of health care facilities [10, 16, 17], direct and indirect cost of services, availability of transport or distance to health facilities [10, 11], household wealth index [6], staff attitude, quality of services, and poor referral linkage influence [15]utilization of skilled birth attendants in low-income countries [6]. In sub-Saharan African countries inequality gaps in the utilization of skilled birth attendants are mostly driven by residency area [16], and maternal education [3, 17]. Mothers living in rural areas and with lower educational levels are less likely to utilize skilled birth attendants as compared to their counterparts living in urban areas and with better educational attainment.

Although monitoring trends and inequality gaps are essential for better planning of services and prioritizing areas of target that are not often done systematically in low-income countries. This study examined the trends in skilled birth attendant national coverage and the inequality gaps in Ethiopia using the Demographic and Health Surveys (DHS) data. Its findings will provide empirical evidence that guides health actions and ensure equitable access to the skilled birth attendant in the country.

## Method

### Study setting

Ethiopia has the second largest population in Africa with an estimated population of 114.9 million in 2020 [16]. The country has a four-tier health care system with a broad base primary health care unit (PHCU) serving the majority of the population. Skilled birth attendant services are given free of charge in all public health facilities with a reasonably functioning referral chain. Ethiopia has expanded free delivery services greatly along with increased demand creation campaigns including what is referred to in the country as the ‘pregnancy conference’ [17, 18].

### Study design and population

This study was based on multiple EDHS surveys (cross-sectional surveys) conducted in the period 2000–2019. The surveys were conducted at approximately 5 years intervals. The study population constitutes women in the reproductive age group, 15–49 years of age, who gave at least one live birth within five years preceding the surveys. A demographic health survey (DHSs) is a standardized and nationally representative cross-sectional survey, it has a standard protocol for selecting study populations [19].

### Sampling method

The DHS typically follows a two-stage stratified cluster sampling procedure to select a nationally representative sample proportionate to the population size of each regional state in the country. The sampling frame was prepared for each round based on the most recent population and housing census. The lowest sampling unit is an Enumeration Area (cluster), which is a geographic area consisting of several dwelling units which served as a counting unit for the census. Enumeration Areas (EAs) were selected using probability proportional to population size. A complete household listing was carried out in all of the selected EAs. In each EA, a fixed number of households per cluster were selected with an equal probability of selection from each newly prepared household list. All women aged 15–49 in the selected households were included in the survey [19–22].

### Data collection

Data were collected using a structured questionnaire consisting of several modules including household and women modules that are relevant to this study [16]. The data were collected through face-to-face interviews with eligible women aged 15–49 who gave birth within five years preceding the survey at home by trained data collectors. If the woman had more than one child in the five years preceding the survey, information on the use of delivery assistance was collected for the last birth [16, 23]. The data for this analysis were accessed by requesting the DHS program website (http://dhsprogram.com/data/available-datasets.cfm) after explaining the purpose of this study. The dependent variable of this study is skilled birth attendant coverage and the main independent variables were household wealth status, women’s education, and residency area.

### Data analysis

The national coverages of skilled birth attendants were calculated by dividing the number of women who reported having skilled delivery by the total number of pregnant women in each survey. The projected coverage for 2025 was derived by calculating a smoothed yearly coverage and compared with the target set for 2025 in HSTP II. The annual increment between surveys was calculated by subtracting the coverage of the last survey from the current survey and then dividing it by the number of years between the two surveys [24].

The equality analysis was done for household wealth quintiles (lowest or poorest, second, middle, fourth, highest or richest), maternal education (No education, Primary school, and Secondary school and above), and place of residence (rural/urban) using the WHO HEAT model [25].

The Toolkit is a software application that facilitates the assessment of health inequalities by using disaggregated data and summary measures through visualized interactive graphs, maps, and tables. The relative concentration index was performed for household wealth status and maternal education and a graph of the urban–rural ratio was done for a place of residency.

The inequality analysis was done for the year 2016 since the data from 2019 was not publicly available.

Study variables were re-coded to meet the desired classification. To overcome the unbalanced distribution of regional samples for national estimates, sampling weights were used during analysis where the inverse probabilities of selection for each observation allow us to reconfigure the sample as if it was a simple random draw of the total population and hence yield accurate population estimates for the main parameters of interest. It also provides a measure of how many individuals a given sampled observation represents in the population.

After searching for literature, the following nine independent variables were included in the analysis namely: Maternal education, wealth status, residence, maternal age, partner’s level of education, number of children, distance to a health facility, maternal occupation, and husband occupation. These independent variables were selected due to their positive association with skilled birth attendance [19, 26, 27].

All analyses were done using Stata version 14. Inferential analysis was used to examine the relationship between the independent variables and the outcome variable. Specifically, binary logistic regression was conducted. All results of the binary logistic analyses were presented as odds ratios (ORs), with 95% confidence intervals (CIs). Variables that show a statistically significant association (*p* < 0.05) at the bivariate level were further analyzed at the multivariate level by logistic regression. All the variables were included in the multivariate model once they were significantly associated at the bivariate level. This is because these variables showed an influence on the outcome variable and there is a need to identify whether each has been confounded by another variable or not. The adjusted odds ratio (AOR) was used to determine the presence of an association between the dependent and independent variables for which 95% CI was determined. The final regression model was tested for fitness using the Pearson goodness of fit test.

## Result

### Trend analysis

**Fig. 1:**
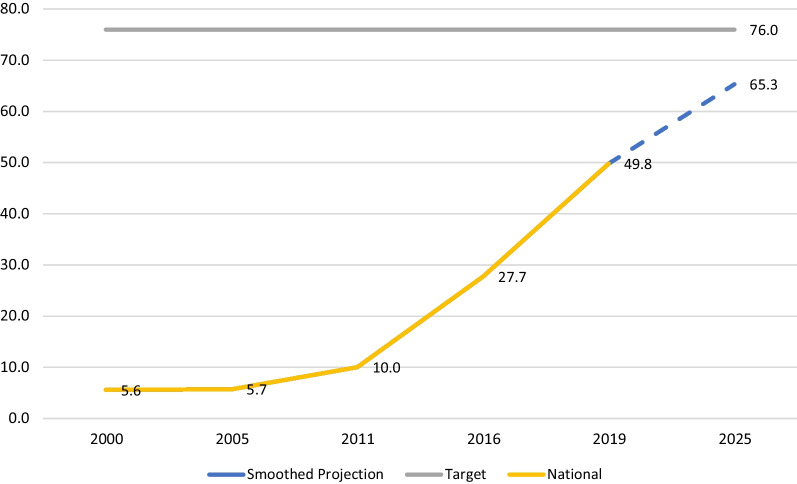
National Trend for skilled birth attendant Coverage is projected for 2025 based on the smoothed average and the most recent trend (2019). Ethiopia 2000–2019

The skilled birth attendant coverage showed a significant and steady increase from 5.6% in 2000 to 49.8 in 2019. If the country continues to move forward at the current pace the 2025 projection will be 94% which is more than the set target. The smoothed average projections indicate 65.3% coverage which would not be enough to achieve the target set for 2025 coverage (Fig. 1).

### Inequality gap analysis

**Fig. 2:**
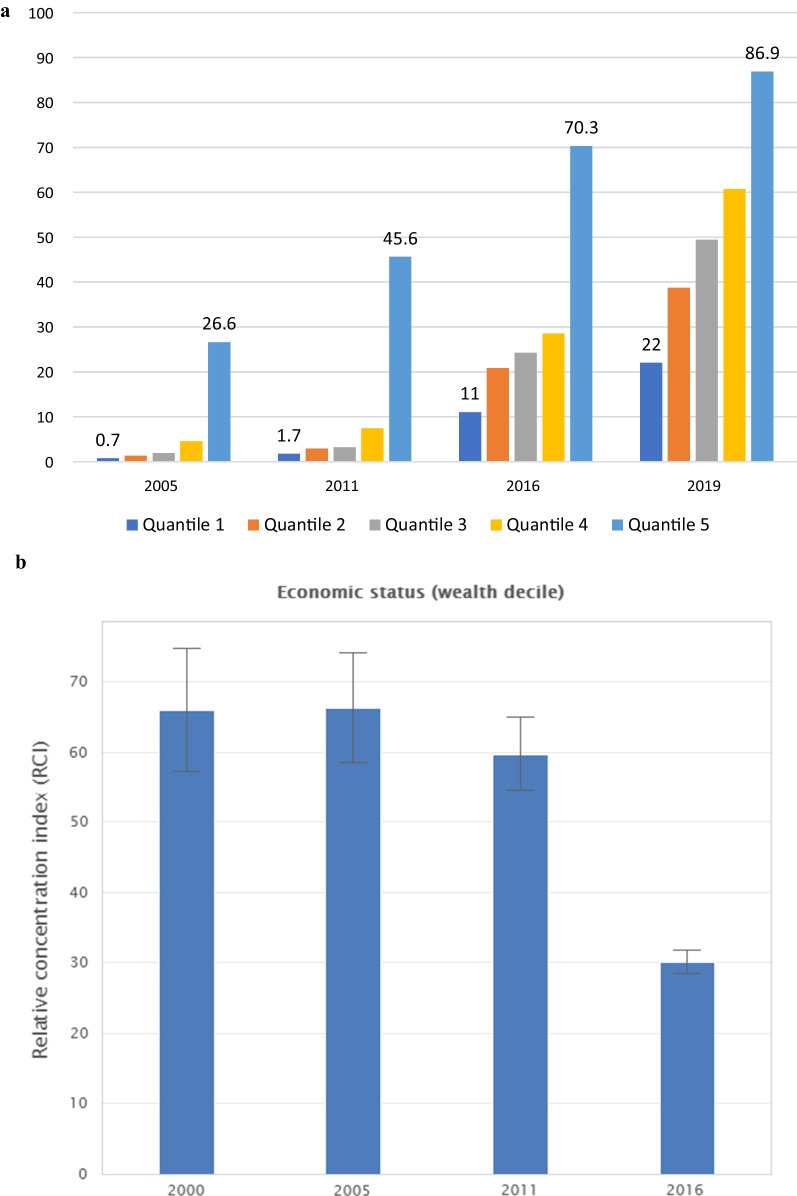
Skilled Birth Attendance coverage inequality among wealth groups in Ethiopia. **a** Coverage gaps between strata 2005–2019 **b** Relative Concentration index 2000–2016

The inequality gap analysis indicated a significant decrease in the gap between the wealth quantiles; the ratio between the highest and the lowest wealth quantile which was 38 in 2005 decreased to 3.95 in 2019 (Fig. 2a). The relative concentration index also showed a similar pattern, a closing of the inequality gap, with a significant and huge reduction in 2016 (Fig. 2b.)

**Fig. 3:**
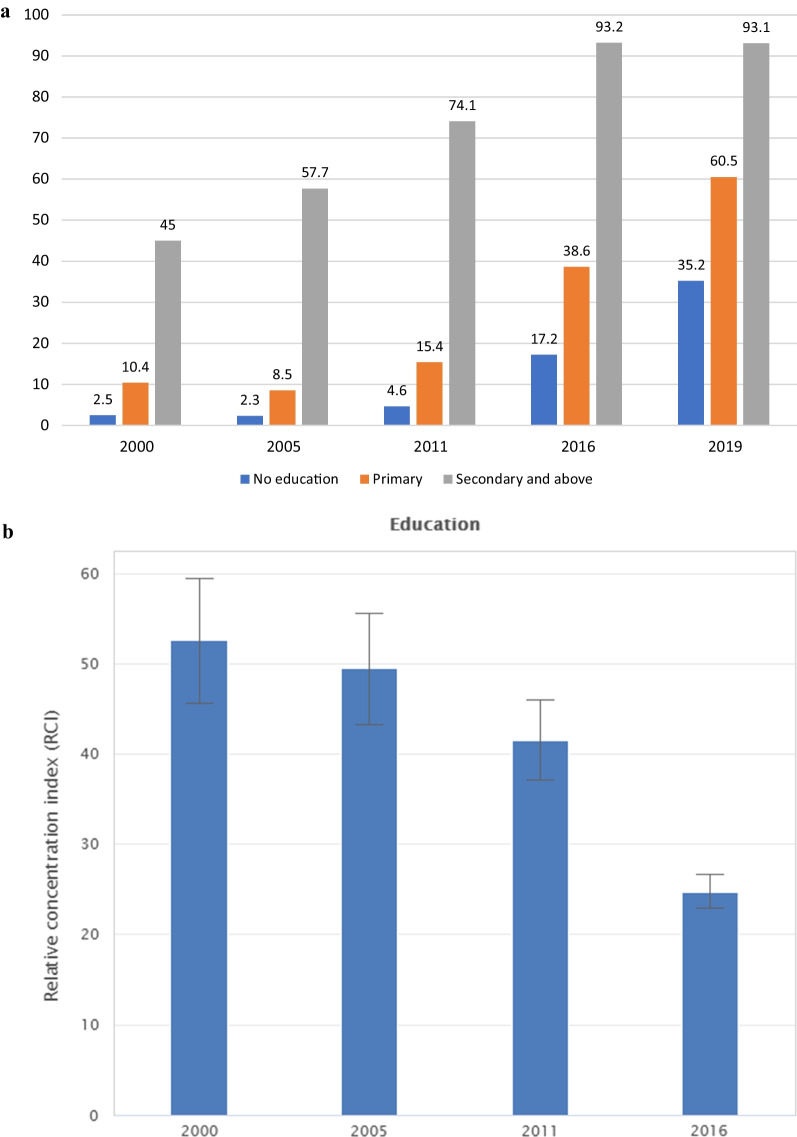
Skilled Birth Attendance coverage inequality among education groups in Ethiopia. **a** Coverage gaps between strata 2000–2019 **b** Relative Concentration index 2000–2016

**Fig. 4:**
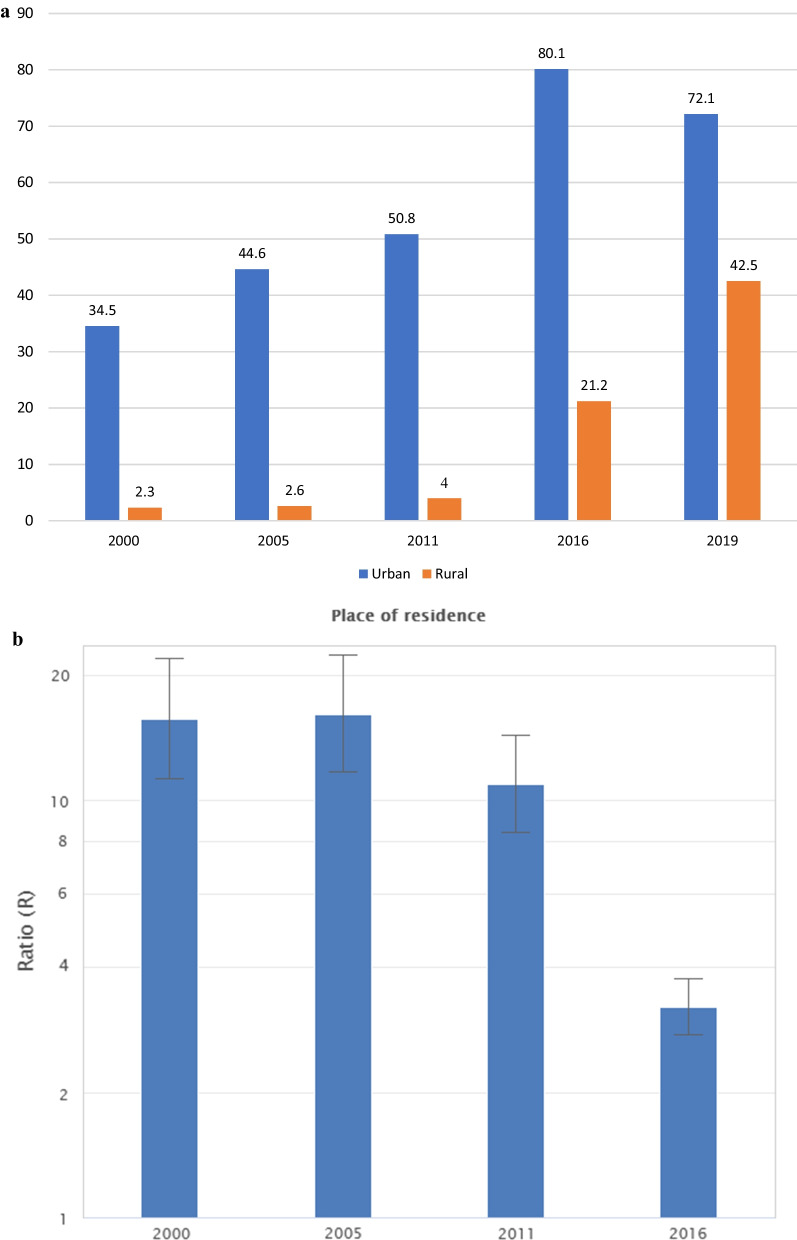
Skilled Birth Attendance coverage inequality among the place of residency in Ethiopia. **a** Coverage gaps between strata 2000–2019 **b** Ratio 2000–2016

The inequality gaps in maternal education and place residency also showed a significant reduction in the gaps between those who attend higher education and lower education (Fig. 3a) and between urban and rural residents (Fig. 4a). The ratio between the highest and lowest strata decreased from 18 to 2.6 between 2000 and 2019 for maternal education and from 15 to 1.7 between urban and rural residents. The relative concentration index showed a significant reduction in 2016 for both maternal education (Fig. 3b) and residency area (Fig. 4b).

### Determinants of skilled delivery

In this study, both maternal and husband educational status, place of residence, household wealth, maternal age, and the number of children were independent factors associated with the utilization of SBA at birth after adjusting the effect of other variables. The odds of utilizing skilled birth attendance were greater in women who have completed at least primary level education [AOR = 1.61 95%CI (1.33, 1.95)] and in the height wealth quantile [AOR = 3.46 95%CI (1.8, 4.31)] and living in urban area [AOR = 3.53 95%CI (1.88, 6.64)] (Table 1). Using the Pearson goodness of fit test, the model was reasonably fit with the *p*-value of 0.51.

**Table 1:** Bivariate and Multivariate Logistic Regression for determinants of SBA utilization in Ethiopia, 2016 EDHS (n = 10,641)

Characteristics	Skilled birth attendance		Bivariate analysis		Multivariable analysis	
	Yes N (%)	No N (%)	Crude OR (95% CI)	*p*-value	Adjusted OR (95% CI)	*p*-value
*Maternal educational status*						
No education	1355 (17.23)	5448 (82.77)	1		1	
Primary	1259 (38.6)	1419 (61.37)	3.02 (2.55, 3.59)	0.001	1.61 (1.33, 1.95)	0.001
Secondary & above	933 (83.5)	192 (16.5)	24.33 (16.55, 35.77)	0.001	2.8 (1.8, 4.31)	0.001
*Wealth index combined*						
Poorest	520 (11)	3473 (89)	1		1	
Poorer	471 (20.8)	1311 (79.1)	2.12 (1.55, 2.88)	0.001	2.06 (1.52, 2.78)	0.001
Middle	434 (24.2)	1032 (75.8)	2.58 (1.89, 3.52)	0.001	2.22 (1.66, 2.96)	0.001
Richer	467 (28.5)	841 (71.5)	3.21 (2.34, 4.4)	0.001	2.24 (1.66, 3.04)	0.001
Richest	1655 (70.3)	437 (29.7)	19.06 (12.95, 28.05)	0.001	3.46 (2.25, 5.30)	0.001
*Type of residency*						
Rural	1971 (21.2)	6696 (75.8)	1		1	
Urban	1576 (80.1)	398 (19.9)	14.9 (9, 24.7)	0.001	3.53 (1.88, 6.64)	0.001

The model was adjusted for maternal age, distance to the health facility, husband and maternal occupation, number of children, and husband education

## Discussion

This study shows that the national Skilled Birth Attendance coverage has been steadily increasing since 2000, significantly more in recent years. The inequality analysis showed the inequality gaps are closing between the advantaged (wealthier, educated, and urban residents) and disadvantaged (poorest, uneducated, and rural residents) groups.

Despite the increased coverage, the achievement is not up to par with the target set by the Health Sector Transformation Plan II for the year 2020 [24] and also for the 2025 target. In the most recent DHS period, the achievement was the largest ever; from 27.7% in 2016 to 49.8% in 2019 [23, 28]. That achievement can be explained by the increasing number of health facilities especially in rural areas and by the increasing availability of the health workforce at the lower tier of the health system [29].

In this study, Women who attend primary plus education level were 1.6 times more likely than those mothers with no formal education in utilizing SBA at delivery and the finding was also consistent with other studies conducted in different parts of Ethiopia and Africa [1, 19, 20]. This is because educated women have better capabilities and confidence to make informed decisions about their health as they are aware of pregnancy-related complications and available health care services, and they are more likely to be financially stable than those who are not educated [1, 30–34]. Having an educated husband was also associated with a better chance of receiving skilled birth attendant services, which is similar to other studies conducted in sub-Saharan Africa [27].

Women who belong to the highest wealth quintile were 3.46 times more likely than women in the poorest wealth quintile in utilizing SBA at delivery. This could be related to a lack of support for receiving SBA due to another household/family responsibilities among the poorest or related to financial challenges that may be needed to transport the mother to the health facilities and pay for additional supplies that are needed during the delivery process, despite skilled birth delivery services being given free of change in public health facilities mothers may be asked to buy supplies that are not available at the health center at the time of delivery [27, 35].

Women living in urban areas were 3.53 times more likely to utilize SBA than women in rural areas. Women living in urban areas have much easy access to skilled birth attendance compared to rural areas due to the proximity of the health facilities and better availability of transportation [36–38]. The steady increment in the trend of skilled birth attendant utilization in a rural areas, a bit lower than in urban areas, could be explained by the expansion of health facilities and the introduction of the health extension program in rural areas [29].

The findings of this study indicated utilization of skilled birth attendants requires further efforts to sustain the gains so far and achieve the target set for the year 2025. Efforts may include adapting, expanding, and evaluating interventions that are more appropriate to the context such as the use of a simple motorized tricycle emergency obstetric transport system (bajaj ambulances), and expanding maternity waiting homes to overcome distance and transportation challenges [26] Additional strategies may be required to cope with unexpected health emergencies such as COVID-19 that can potentially affect utilization of Skilled birth attendance services. For instance, some public health facilities were designated to handle only COVID-19-related services and stopped the provision of non-COVID-19-related health services [39]. Women may also refrain from using services due to fear of contracting the virus and travel restrictions [40].

Despite using large representative data and rigorous analytical approaches recall bias was the limitation of this study. Also, due to the cross-sectional nature of the data, we cannot establish the temporal relationship between the independent and dependent variables.

## Conclusion

The utilization of skilled birth attendants in Ethiopia has remarkably increased in the last two decades. Despite the still lingering inequality gaps in household wealth status, women’s education, and residency area, the country was able to significantly narrow the gaps between the advantaged and disadvantaged groups. However, there is still a gap between the advantaged and disadvantaged groups. Therefore, the country needs to intensify its efforts to eliminate these inequality gaps by expanding access for all mothers to appropriate health service outlets. Specific interventions like involving and coordinating different multisectoral efforts are also needed to improve the economic and educational status of women which in long run will affect their attendant preference during birth. Giving priority to further reaching the poorest and those living in rural areas is also needed to achieve national and international targets.

## Data Availability

Data for this study is available at the following website: http://dhsprogram.com/data/available-datasets.cfm.

## Abbreviations

AOR: Adjusted odds ratio
COVID -19: Coronavirus disease 2019
DHS: Demographic and health survey
EA: Enumeration areas
EDHS: Ethiopian demographic and health survey
FIGO: International federation of gynecology and obstetrics
HEAT: Health equity assessment toolkit
HSTP II: Health sector transformation plan
HEW/HAD: Health extension workers
ICM: International confederation of midwives
LMIC: Low-and middle-income countries
MCH: Maternal and child health
MMR: Maternal mortality ratio
MOH: Ministry of health
PHCU: Primary health care unit
RCI: Relative concentration index
SBA: Skilled birth attendant
STATA: Statistical software for data science
WHO: World Health Organization
